# Expanding the Use of PARP Inhibitors as Monotherapy and in Combination in Triple-Negative Breast Cancer

**DOI:** 10.3390/ph14121270

**Published:** 2021-12-06

**Authors:** Mariya Yordanova, Audrey Hubert, Saima Hassan

**Affiliations:** 1 Faculty of Medicine and Health Sciences, Université de Sherbrooke, Sherbrooke, QC J1K 2R1, Canada; Mariya.Yordanova@usherbrooke.ca; 2 Faculty of Medicine, Université de Montréal, Montréal, QC H3C 3T5, Canada; audrey.hubert.chum@ssss.gouv.qc.ca; 3 Centre de Recherche du Centre Hospitalier de l’Université de Montréal (CRCHUM), l’Institut de Cancer de Montreal, Montreal, QC H2X 0A9, Canada; 4 Division of Surgical Oncology, Department of Surgery, Centre Hospitalier de l’Université de Montréal (CHUM), Montreal, QC H2X 0C1, Canada

**Keywords:** triple-negative breast cancer, PARP inhibitors, combination therapy, predictive biomarkers

## Abstract

Triple-negative breast cancer (TNBC) is the most aggressive subtype of breast cancer, and is known to be associated with a poor prognosis and limited therapeutic options. Poly (ADP-ribose) polymerase inhibitors (PARPi) are targeted therapeutics that have demonstrated efficacy as monotherapy in metastatic *BRCA*-mutant (BRCA^MUT^) TNBC patients. Improved efficacy of PARPi has been demonstrated in BRCA^MUT^ breast cancer patients who have either received fewer lines of chemotherapy or in chemotherapy-naïve patients in the metastatic, adjuvant, and neoadjuvant settings. Moreover, recent trials in smaller cohorts have identified anti-tumor activity of PARPi in TNBC patients, regardless of *BRCA*-mutation status. While there have been concerns regarding the efficacy and toxicity of the use of PARPi in combination with chemotherapy, these challenges can be mitigated with careful attention to PARPi dosing strategies. To better identify a patient subpopulation that will best respond to PARPi, several genomic biomarkers of homologous recombination deficiency have been tested. However, gene expression signatures associated with PARPi response can integrate different pathways in addition to homologous recombination deficiency and can be implemented in the clinic more readily. Taken together, PARPi have great potential for use in TNBC patients beyond BRCA^MUT^ status, both as a single-agent and in combination.

## 1. Introduction

Breast cancer in women is the most common malignancy worldwide [1]. Triple-negative breast cancer (TNBC) is the most aggressive subtype, accounting for 15–20% of all breast cancer patients [2]. Characterized by the absence of estrogen receptor (ER), progesterone receptor (PR), and human epidermal growth factor receptor-2 (HER2) overexpression, TNBCs are heterogeneous tumors with diverse clinical outcomes [2]. Germline mutations in *BRCA1/2* (gBRCA^MUT^) are frequently identified in TNBC patients, ranging from 11–20% [3,4,5,6], in comparison to 5–7% [7,8,9] of all breast cancers. TNBCs are known to have frequent tp53 mutations (83%), and aneuploid (abnormal chromosome number) rearrangements (80%) [10,11]. TNBC is further subdivided into six molecular subtypes including two-basal-like, an immunomodulatory, a mesenchymal, a mesenchymal stem-like, and a luminal androgen receptor subtype [12]. Clinically, TNBCs have a predilection to develop distant metastasis to visceral organs and the central nervous system [2,13]. TNBC patients have the poorest clinical outcomes in comparison to endocrine-sensitive and HER2-positive breast cancer with a three-fold higher rate of distant recurrence and mortality within the first five years following initial diagnosis [14].

**Figure 1:**
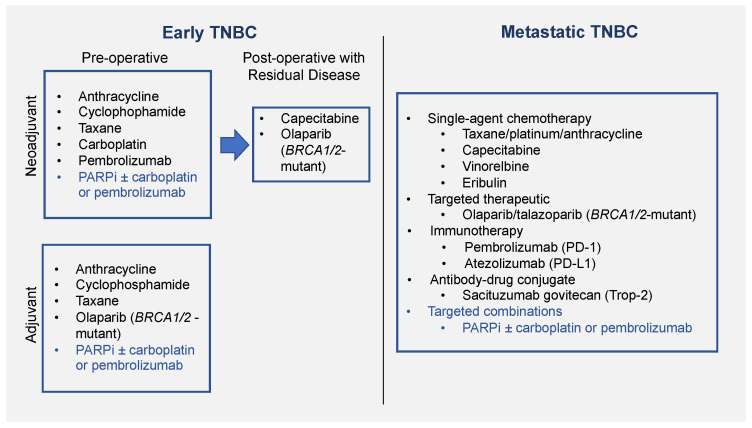
Current and proposed treatment options in TNBC. On the left are treatment options for early breast cancer, and on the right are treatment options for metastatic breast cancer. Current treatment options are in black, and future potential treatment options are in blue. PARPi, PARP inhibitors.

TNBC patients do not benefit from antihormonal or anti-HER2 therapy and receive conventional chemotherapy including doxorubicin (adriamycin), cyclophosphamide, and paclitaxel [15,16] (Figure 1). Platinum agents such as carboplatin are of increasing interest in gBRCA^MUT^, and more recently in *BRCA*-wild type (BRCA^WT^) patients [15,17]. Paradoxically, TNBC patients tend to have a higher pathologic complete response (pCR) rate in the neoadjuvant setting (35–50%) versus other breast cancer subtypes, but still have poorer outcomes [18,19]. This may be due to the fact that TNBC patients with residual disease have a shorter overall survival (OS) than other breast cancer subtypes [18]. Capecitabine, an oral chemotherapeutic, is also offered to TNBC patients with residual disease following neoadjuvant chemotherapy [20].

Two promising treatment strategies that have recently emerged for TNBC patients are immune-checkpoint inhibitors (atezolizumab and pembrolizumab) and an antibody-drug conjugate (sacituzumab govitecan). Atezolizumab, a programmed death (PD)-ligand (L)1 inhibitor, improved progression-free survival (PFS) and OS when combined with nab-paclitaxel in metastatic TNBC patients with PD-L1–positive tumors [21]. Pembrolizumab, a PD-1 inhibitor, was also associated with an improvement in pCR and event-free survival when administered in combination with four chemotherapeutic agents in the neoadjuvant setting [22,23]. However, some of the challenges of immunotherapy include predicting immune-related adverse events as well as improving response rates given that a spectrum of response patterns is seen across patients [16,24]. Sacituzumab govitecan targets Trop-2 (antitrophoblast cell-surface antigen 2) and allows for intracellular internalization of SN-38, a metabolite of a chemotherapeutic irinotecan. In metastatic TNBC patients, sacituzumab govitecan demonstrated a significant improvement in PFS and OS, but myelosuppression and diarrhea occurred more frequently in comparison to standard of care chemotherapy [25].

Poly (ADP-ribose) polymerase inhibitors (PARPi) are orally available therapeutic agents that demonstrated improved efficacy and less toxicity than standard single-agent chemotherapy in metastatic gBRCA^MUT^ patients [26,27]. PARPi target the PARP1/2 enzymes and have two main mechanisms of action: synthetic lethality, and PARP-DNA trapping [28,29]. In synthetic lethality, PARPi are catalytic inhibitors that prevent the release of PARP1 from DNA, causing stalling of the replication fork. When *BRCA1/2* is mutated, alternative repair mechanisms lead to complex chromatid rearrangements and cell death [30,31]. Trapped PARP-DNA complexes result in DNA lesions that are not bypassed by replication forks and induce cytotoxicity [29,32,33]. PARPi demonstrate similar catalytic activity but differ in their PARP-DNA trapping. PARP-DNA trapping capacity has been shown to correlate with cytotoxic potency. From lowest to highest potency, clinical PARPi are ranked as follows: veliparib, olaparib, which is similar to rucaparib, niraparib, and talazoparib [34,35].

Olaparib and talazoparib were approved as monotherapy in metastatic TNBC or hormone-refractory gBRCA^MUT^ patients by the FDA and Health Canada [36,37,38,39]. However, emerging studies are suggesting a broader utility of PARPi beyond gBRCA^MUT^ patients. Indeed, there is a role for PARPi amongst tumors that demonstrate BRCAness, i.e., tumors that share many clinicopathological features as BRCA^MUT^ tumors without having the mutation [40,41]. In this review article, we focus on the evolution of PARPi in the field of breast cancer, particularly in metastatic TNBC as well as in the neoadjuvant and adjuvant settings. In addition, we summarize the studies evaluating PARPi in combination with chemotherapy, immunotherapy, and targeted therapy, as well as the use of PARPi in the elderly population, to elucidate the clinical potential of PARPi in TNBC. Furthermore, we provide a comprehensive overview of the recent advancements of predictive biomarkers that have been used to identify a patient subpopulation of TNBCs that will best respond to PARPi.

## 2. PARPi as Monotherapy

### 2.1. Use in Metastatic Setting

PARPi have demonstrated an improved efficacy in recent years in metastatic breast cancer, summarized in Table 1. Although phase I trials have focused on safety in smaller cohorts of patients, anti-tumor activity was nonetheless observed amongst gBRCA^MUT^ patients. In 2009, Fong et al. evaluated olaparib in a phase I trial of which 71% of the patients received ≥3 previous treatment regimens [42]. The maximum tolerated dose (MTD) was established at 400 mg twice daily. A partial or complete radiological response was observed in 47.4% of the BRCA^MUT^ patients (including breast, ovarian, or prostate cancers), but not in any BRCA^WT^ patients. In 2017, a phase I study established the MTD for talazoparib as 1.0 mg/day [43]. In BRCA^MUT^ patients who received up to 6 lines (median 2) of prior chemotherapy, the objective response rate (ORR) was 50% and 42% for breast and ovarian cancer patients, respectively. The recommended phase II dose for veliparib as monotherapy was identified at 400 mg twice a day [44]. Despite patients receiving up to 14 previous treatment regimens, the ORR in BRCA^MUT^ patients treated at all doses was 23%, but only 4% amongst BRCA^WT^ patients [44,45].

Three phase II trials evaluated the safety and efficacy of olaparib in patient cohorts of different cancer types. First, Tutt and colleagues performed a non-randomized proof-of-concept trial to evaluate two dosing regimens of olaparib, 400 mg and 100 mg twice daily, in advanced BRCA^MUT^ breast cancers [46]. Patients had received up to 5 previous chemotherapy regimens (median 3). The ORR was 41% (95% confidence interval (CI), 25–59) in the cohort assigned to 400 mg twice daily, and 22% (95% CI, 11–41) in the cohort receiving 100 mg twice daily, demonstrating a dose-dependent response to PARPi in BRCA^MUT^ breast cancers [46].

Second, Gelmon et al. evaluated the efficacy of olaparib in a phase II trial in 90 BRCA^MUT^ and BRCA^WT^ patients with either ovarian carcinoma or TNBC [47]. Patients received up to 10 prior chemotherapy regimens, with more than 70% of the breast cancer participants exposed to ≥3 lines of chemotherapy. While olaparib treatment achieved an ORR of 41% (95% CI, 22–46) in BRCA^MUT^ and 24% (95% CI, 14–38) in BRCA^WT^ ovarian cancer patients, no confirmed objective responses were reported for the breast cancer cohort, regardless of *BRCA*-mutation status [47]. However, this study had several limitations that could potentially account for the differences in results between the ovarian and breast cancer cohorts. The breast cancer cohort comprised of 26 patients, of which only 10 patients were BRCA^MUT^. Hence, a small sample size renders the interpretation of the absence of an objective response difficult. The study design was non-blinded and non-randomized. Moreover, the median PFS in the ovarian and breast cancer cohorts were 219 days (range 110–273) and 54 days (range 51–106), respectively, suggesting that the breast cancer patients were at a very advanced stage.

Third, Kaufman et al. investigated the impact of olaparib monotherapy in a phase II trial in advanced solid cancer patients with gBRCA^MUT^ [48]. The breast cancer cohort consisted of 62 patients, and eligibility criteria included ≥3 prior chemotherapy regimens for metastatic disease. The tumor response rate was 12.9% (95% CI, 5.7–23.9) and the median PFS was 3.7 months. The response rate reported here is significantly lower compared to the 41% identified in the cohort treated with the identical dose in the study conducted by Tutt et al. [46]. However, 100% of the breast cancer patients in Kaufman et al.’s trial received ≥3 chemotherapy regimens for metastatic disease, of which 68% received prior platinum therapy. In contrast, 21% of the patients received ≥3 prior chemotherapy regimens for metastatic disease, and 22% received prior platinum therapy in the cohort receiving 400 mg of olaparib in Tutt et al.’s study. Altogether, the phase I and II trials demonstrated potential for PARPi in BRCA^MUT^ breast cancer patients, which warranted further investigation in larger, randomized controlled trials. 

**Table 1:** Clinical trials with PARP inhibitors as monotherapy in the metastatic setting.

First Author Study Name	Year of Study	No. of Participants (BC Patients)	Type of Study	Median/Mean No. of Prior Chemotherapy Regimens (Range)	Comparative Arms		Patient Population	Outcome (Objective Response Rate, Progression Free Survival)
					PARPi	Comparative Agent/ Standard Chemotherapy		
Fong, P.C. et al. [42]	2009	60 (9 BC)	Phase I	28% ≤ 2 lines * 18%—3 lines * 53% ≥ 4 lines *	Olaparib Cohort 1: 10 mg/day to 600 mg twice daily Cohort 2: 200 mg twice daily	None	BRCA^MUT^: N = 22 BRCA^WT^: N = 38	ORR in all BRCA^MUT^: 47.4% No objective response in BRCA^WT^
de Bono, J. et al. [43]	2017	110 (20 BC)	Phase I	2.5 (0–13)	Talazoparib Part 1: 0.025 to 1.1 mg/day Part 2: 1.0 mg/day	None	Part 1: DNA repair deficiency; Part 2: gBRCA^MUT^	ORR in BRCA^MUT^ in breast cancer: 50% PFS in breast cancer: 34.6 weeks
Puhalla S et al. [44] Pahuja S et al. [45]	2014	98 (40 BC)	Phase I	gBRCA^MUT^: 6 (1–14) * BRCA^WT^: 4 (1–12) *	Veliparib 50–500 mg twice daily	None	gBRCA^MUT^: N = 70 BRCA^WT^: N = 28	ORR in all BRCA^MUT^: 23%, breast BRCAMUT: 29% ORR in all BRCA^WT^: 4%, breast BRCAMUT: 5%
Tutt, A. et al. [46]	2010	54 (54 BC)	Phase II, non-randomized sequential-cohort	Cohort 1: 3 (1–5) Cohort 2: 3 (2–4)	Olaparib Cohort 1: 400 mg twice daily Cohort 2: 100 mg twice daily	None	gBRCA^MUT^	ORR in cohort 1: 41%, cohort 2: 22% PFS in cohort 1: 5.7 months; PFS in cohort 2: 3.8 months
Gelmon, K.A. et al. [47]	2011	91 (26 BC)	Phase II, non-randomized	3 (1–7)	Olaparib capsule 400 mg twice daily	None	BRCA^MUT^: N = 27 BRCA^WT^: N = 63	ORR in ovarian cancer: BRCA^MUT^ 41%, BRCA^WT^ 24%; breast cancer: BRCA^MUT^ 0% BRCA^WT^ 0% PFS in ovarian cancer: BRCA^MUT^ 7.4 months, BRCA^WT^: 6.4 months; breast cancer: BRCA^MUT^ 3.6 months, BRCA^WT^: 1.8 months
Kaufman, B. et al. [48]	2015	298 (62 BC)	Phase II, single-arm, non-randomized	BC cohort: 4.6 (3–11)	Olaparib capsule 400 mg twice daily	None	gBRCA^MUT^	Response rate for all: 26.2%; breast cancer 12.9% PFS in breast cancer 3.7 months
Robson, M.E. et al. [26,49] OlympiAD	2017	302 (302 BC)	Phase III, randomized	≤2 lines	Olaparib tablet 300 mg twice daily	Capecitabine, eribulin, or vinorelbine	gBRCA^MUT^ HER2-negative	ORR 59.9% vs. 28.8% (olaparib versus standard chemotherapy) PFS 7.0 months vs. 4.2 months (olaparib versus standard chemotherapy)
Litton J.K. et al. [27] EMBRACA	2018	431 (431 BC)	Phase III, randomized	≤3 lines	Talazoparib 1 mg once daily	Capecitabine, eribulin, gemcitabine, or vinorelbine	gBRCA^MUT^ HER2-negative	ORR 62.2% vs. 27.2% (olaparib versus standard chemotherapy) PFS 8.6 months vs. 5.6 months (olaparib versus standard chemotherapy)
Tung N.M. et al. [50] TBCRC 048	2020	54 (54 BC)	Phase II, non-randomized	1 (0–4)	Olaparib tablet 300 mg twice daily	None	Cohort 1: Germline mutation in HR-related gene (not gBRCA1/2) Cohort 2: Somatic mutations in same genes (including BRCA1/2)	ORR in all cohort 1, 33%; gPALB2^MUT^ 82%; all cohort 2, 31%; sBRCA^MUT^ 50% PFS for gPALB2^MUT^, 13.3 months; sBRCA^MUT^ 6.3 months

Abbreviations: BC, breast cancer. * Previous treatment regimen.

OlympiAD was a phase III open-label randomized controlled trial, wherein olaparib was compared with standard chemotherapy, treatment of physician’s choice (TPC), in patients with gBRCA^MUT^ and HER2-negative metastatic breast cancer [26]. Patients were eligible if they received ≤2 lines of prior chemotherapy regimens. Three hundred and two patients were randomized to receive either olaparib tablets (300 mg twice daily) or TPC (capecitabine, vinorelbine, or eribulin). Patients treated with olaparib in comparison to TPC demonstrated an improvement in median PFS (7.0 months vs. 4.2 months, respectively; hazard ratio (HR) for disease progression or death, 0.58; 95% CI, 0.43–0.80; *p* < 0.001), and an ORR of 59.9% (95% CI, 52.0–67.4) versus 28.8% (95% CI, 18.3–41.3) for the standard chemotherapy arm [26]. A prespecified subgroup analysis demonstrated a significant improvement in OS amongst those patients who had received no prior chemotherapy while comparing patients who received olaparib versus TPC (22.6 months versus 14.7 months; HR, 0.51; 95% CI, 0.29–0.90; *p* = 0.02). However, in patients who did receive prior chemotherapy (2nd or 3rd line), there was no statistically significant difference in OS between olaparib and standard treatment (18.8 months versus 17.2 months; HR, 1.13; 95% CI, 0.79–1.64) [49]. Adverse events during olaparib treatment were commonly low grade, with a rate of grade 3 or higher adverse events of 36.6%, in comparison to 50.5% for patients with TPC. Adverse events that were more common in the olaparib arm were anemia, nausea, vomiting, fatigue, headache, and cough, but overall, manageable by supportive treatment or dose modification.

Similarly, the use of talazoparib as monotherapy was evaluated in the EMBRACA trial in gBRCA^MUT^ patients with locally advanced or metastatic breast cancer [27]. The phase III, open-label, randomized trial compared talazoparib (1 mg once daily) to standard single-agent chemotherapy (capecitabine, eribulin, gemcitabine, or vinorelbine) in 431 patients. Ninety-five percent of the patients received ≤2 lines of prior chemotherapy regimens. Patients receiving talazoparib demonstrated a significantly increased median PFS compared to those receiving standard therapy (8.6 months vs. 5.6 months, respectively; HR, 0.54; 95% CI, 0.41–0.71; *p* < 0.001). A higher ORR was observed in patients treated with talazoparib compared to chemotherapy (62.6% versus 27.2%; odds ratio (OR), 5.0; 95% CI, 2.9–8.8; *p* < 0.001) [27], with a superior mean duration of response of 5.4 months in patients who received talazoparib, in comparison to 3.1 months in patients who received standard therapy. As for safety assessment, hematologic adverse events—primarily anemia, fatigue, and nausea, were more common among patients randomized to talazoparib, while non-hematologic adverse events were comparable between both groups, demonstrating a tolerable side-effect profile for talazoparib [27].

A meta-analysis of the OlympiAD and EMBRACA studies further demonstrated that PARPi agents significantly delayed time to clinically meaningful quality of life (QoL) deterioration [50]. In comparison to standard monochemotherapy, the use of single-agent PARPi was associated with a significantly increased PFS and ORR in both clinical trials. The use of PARPi seems to reduce the risk of common side effects of chemotherapy such as neutropenia and any grade palmar-plantar erythrodysesthesia syndrome while increasing the risk of anemia and any grade headache [50].

Recently, Olaparib Expanded, a phase II study, assessed olaparib response in patients with HER2-negative metastatic breast cancer with somatic (s)BRCA^MUT^ or germline/somatic mutations in homologous recombination (HR)–related genes other than *BRCA1/2* [51]. Fifty-four patients were enrolled, of which 76% had ER-positive disease. Olaparib was particularly effective in patients with germline PALB2 mutation (ORR 82%) or sBRCA^MUT^ (ORR 50%), suggesting a plausible role for PARPi beyond gBRCA^MUT^, including TNBC and hormone receptor-positive breast cancer patients [51].

### 2.2. Use in Adjuvant Setting

OlympiA, a phase III, double-blinded, randomized controlled trial, evaluated the role of olaparib as adjuvant therapy in 1836 patients with HER2-negative early breast cancer and gBRCA^MUT^ pathogenic or likely pathogenic variants who had received local treatment and neoadjuvant or adjuvant chemotherapy [52]. Adjuvant olaparib was associated with an increase in 3-year invasive disease-free survival compared to placebo (85.9% versus 77.1%; HR, 0.58; 99.5% CI, 0.41–0.82; *p* < 0.001) as well as an increase in 3-year distant disease–free survival (87.5% versus 80.4%; HR, 0.57; 99.5% CI, 0.39–0.83; *p* < 0.001). Furthermore, olaparib was associated with fewer deaths than placebo (59 and 86, respectively; HR, 0.68; 99% CI, 0.44–1.05; *p* = 0.02), however, a longer follow-up is required to better evaluate the effect on OS. Therefore, one year of adjuvant olaparib was significantly associated with a decrease in recurrence risk and prevented progression to metastatic disease in patients with gBRCA^MUT^ and high-risk early breast cancer. Interestingly, while the OlympiA trial demonstrated that olaparib improved invasive disease-free survival by 8.8% in comparison to control, the CREATE-X trial showed that capecitabine improved invasive disease-free survival by 6.5% in mainly TNBC patients with residual disease [20]. While olaparib demonstrated an improved toxicity profile in the metastatic setting in comparison to TPC including capecitabine [26], a back-to-back randomized comparative trial would be required to truly compare the efficacy and toxicity of the two oral therapeutic agents in the adjuvant setting.

### 2.3. Use in Neoadjuvant Setting

The use of PARPi in the neoadjuvant setting has similarly been an area of interest. Litton et al. first performed a pilot study to evaluate the response to talazoparib in 20 HER2-negative gBRCA^MUT^ patients with operable breast cancer [53]. Patients with stage I to III breast cancer were treated with talazoparib for 6 months followed by definitive surgery and tumors were evaluated for pCR or RCB (residual cancer burden). The RCB-0/pCR rate was 53% (95% CI, 32–73%) and RCB-0/I (no or minimal residual disease) was 63% (95% CI, 41–81%). Efficacy was observed across patients with both *BRCA1* and *BRCA2* mutations, hormone receptor-positive tumors, TNBCs, in addition to chemotherapy-resistant tumors, including metaplastic and inflammatory breast cancers. Subsequently, a phase II neoadjuvant trial was conducted in 112 patients who were treated with talazoparib as a single-agent for more than 20 weeks [54]. In the intention-to-treat population (patients who received ≥1 dose of talazoparib), a pCR rate of 49.2% (95% CI, 36.7–61.6) was identified. Indeed, these results are comparable to those observed with standard chemotherapy in TNBC patients [18]. Therefore, in the neoadjuvant context, as monotherapy, talazoparib can achieve pCR in the gBRCA^MUT^ patient population, and it is plausible that talazoparib can be used to de-escalate the use of chemotherapy.

Importantly, PARPi have also been evaluated in an unselected cohort of primary TNBC patients who did not receive any prior chemotherapy. In the phase II PETREMAC trial, olaparib was administered as a single agent for up to 10 weeks before the administration of chemotherapy in 32 patients. In the unselected TNBC cohort, the ORR was 56.3%, and amongst patients not harboring gBRCA1/2 or gPALB2 mutations, the ORR was 51.9% [55]. Furthermore, the phase II, window-of-opportunity RIO trial was conducted in primary TNBC patients in which rucaparib was administered first, followed by neoadjuvant chemotherapy or surgery [56]. Herein, 43 patients were enrolled, of which 81.4% of patients were not BRCA^MUT^. Rucaparib was associated with >75% decline in circulating tumor DNA (ctDNA) in 58% of TNBC patients. Indeed, the results from the PETREMAC and RIO trials are suggestive that efficacy of PARPi can be observed in both BRCA^MUT^ and BRCA^WT^ TNBC patients, which contrasts with the results observed by Gelmon et al.’s metastatic TNBC cohort that was heavily pre-treated and at a very advanced stage [47]. This is suggestive that the role of PARPi in TNBC needs to be re-evaluated in the chemotherapy-naïve or minimally pre-treated context using larger sample sizes and a randomized study design.

## 3. PARPi as Combination Therapy

### 3.1. PARPi in Combination with Chemotherapy

#### 3.1.1. Use in Metastatic Setting

Several phase I trials have evaluated the combination of PARPi in combination with cytotoxic chemotherapy in the metastatic setting, which included smaller cohorts of breast cancer patients (Table 2). In a phase I/Ib trial with gBRCA^MUT^ breast and ovarian cancer patients, Lee et al. evaluated olaparib plus carboplatin, wherein patients received a median of 5 prior chemotherapy regimens (range, 2–11) [57]. In the first two cycles, olaparib was administered continuously at 100–200 mg twice daily plus carboplatin AUC 3 once every 21 days, followed by intermittent dosing on days 1–7 of olaparib at 400 mg twice daily with carboplatin AUC 3–5 every 21 days for subsequent cycles. Objective responses were observed in 52.4% of patients and rates of grade 3/4 toxicities included 42.2% for neutropenia, 20.0% for thrombocytopenia, and 15.6% for anemia. To better evaluate the impact of the sequencing of the two therapeutics, the same group conducted another phase I/Ib trial in breast and gynecological cancers, which was not restricted to gBRCA^MUT^ patients [58]. Patients were either in a dose-escalation cohort or an expansion cohort with a sequenced administration of either olaparib-first or carboplatin-first. Patients had received a median of 4 prior chemotherapy regimens (range, 1–10). Dose-limiting toxicity was thrombocytopenia and neutropenia, with the MTD of olaparib 200 mg twice daily and carboplatin AUC 4. An ORR of 46% was observed. The rates of grade 3/4 toxicities included 25% for neutropenia, 13% for thrombocytopenia, and 9% for anemia. Subsequent in-vitro experiments demonstrated that pre-treatment with carboplatin increases olaparib clearance by augmenting intracellular olaparib accumulation, suggesting that pre-exposure with carboplatin can improve clinical efficacy.

The combination of talazoparib and carboplatin was evaluated in a phase I trial in patients with advanced solid cancers [59]. Patients received starting doses of talazoparib continuously at 0.75 mg twice daily and carboplatin weekly at AUC 1. Seventy-five percent of the cohort had received ≥3 prior lines of therapy, of which 58% of the patients previously received carboplatin. The observed ORR was 14%. Hematologic toxicities post-cycle 2 necessitated dose delays and/or reductions in all patients. The rates of grade 3/4 toxicities were 13% for fatigue, 63% for neutropenia, 29% for thrombocytopenia, and 38% for anemia. Decreases in neutrophils and white blood cell counts were more pronounced in gBRCA^MUT^ carriers compared to non-carriers. Pharmacokinetic toxicity modeling suggested that the combination of PARPi and chemotherapy may be optimized by pulse dosing (introducing talazoparib-free periods) with particular attention to dosing in the context of gBRCA^MUT^ or BRCA^WT^ patients.

The combination of veliparib and carboplatin was also evaluated in a phase I/II trial with metastatic gBRCA^MUT^ breast cancer patients [60]. In the phase I component, patients received veliparib 50–200 mg twice daily continuously and carboplatin AUC 5–6 on day 1, every 21 days. In the phase II component, patients received veliparib 400 mg twice daily continuously, and upon progression received the combination with carboplatin. The median number of prior chemotherapy regimens was 1 (range, 0–5). For the phase I component, a response rate of 56% was identified, with a PFS and OS of 8.7 and 18.8 months, respectively. For the phase II component, the response rate was 14%, with a PFS and OS of 5.2 and 14.5 months, respectively. Cytopenias leading to dose reductions or delays occurred in 75% of the phase I patients after cycles 1–3. Grade 3/4 toxicities in this phase included 59% thrombocytopenia, 52% neutropenia, and 25% anemia. A phase I study was also conducted with veliparib in combination with carboplatin and paclitaxel in advanced solid malignancies [61]. Here, paclitaxel and carboplatin were administered every 21 days, while veliparib was administered on days 1–7 from cycle 2 and onwards. All patients received ≤3 prior chemotherapy regimens. The observed ORR was 37%. The recommended phase II doses were veliparib 100 mg twice daily, paclitaxel 200 mg/m^2^, and carboplatin AUC 6. Interestingly, an increase in myelosuppression was not observed in cycle 2 with the addition of veliparib, in comparison to cycle 1, suggestive that intermittent dosing can assist with hematologic recovery.

The BROCADE studies compared the efficacy of veliparib in combination with carboplatin/paclitaxel (VCP), versus placebo plus carboplatin/paclitaxel (PCP) in locally recurrent or metastatic gBRCA^MUT^ breast cancer patients [62]. Veliparib was dosed at 120 mg twice daily. During the three-week cycle, veliparib was administered with a 2-day run-in, for a total of 7 days, carboplatin on day 1, and paclitaxel on days 1, 8, 15. The first BROCADE study was a randomized, partially blinded phase II trial consisting of 290 patients. The ORR in the VCP arm was superior to the PCP arm (77.8% versus 61.3%; *p* = 0.027). BROCADE3 was a randomized, double-blind, placebo-controlled phase III trial, which further evaluated VCP versus PCP, with the optional continuation of monotherapy with veliparib if the platinum doublet chemotherapy was discontinued before progression [63]. Five hundred and nine patients were enrolled, of which approximately 50% constituted TNBC. For the entire cohort, the median PFS was 14.5 months in the veliparib-based arm versus 12.6 months in the control arm (HR, 0.71; 95% CI, 0.57–0.88; *p* = 0.0016). Amongst patients who did not receive prior chemotherapy for metastatic disease, patients receiving VCP demonstrated a greater improvement in PFS in comparison to those in the PCP arm (16.6 months versus 13.0 months, respectively; HR, 0.70; 95% CI, 0.54–0.89; *p* = 0.004) with durable benefits present at 2 years and 3 years post-randomization [64]. Once again, we see an enhanced benefit of PARPi response in patients with less prior chemotherapy. The most common grade 3/4 adverse events had similar frequency in the veliparib and chemotherapy arms. In particular, neutropenia occurred in 81% of the veliparib-based arm and 84% in the control arm, anemia in 42% versus 40%, and thrombocytopenia in 40% versus 28%, respectively.

#### 3.1.2. Use in Neoadjuvant Setting

In the neoadjuvant setting, an adaptive randomized controlled trial, the I-SPY 2 TRIAL (Investigation of Serial studies to Predict Your Therapeutic Response with Imaging and molecular AnaLysis 2) evaluated the combination of continuous low-dose veliparib (50 mg twice a day) plus carboplatin in comparison to standard chemotherapy (doxorubicin, cyclophosphamide, and paclitaxel) [65]. Within the TNBC subpopulation, the addition of veliparib plus carboplatin to standard therapy resulted in a significant increase in pCR at 51% (95% Bayesian probability interval [PI], 36–66%), in comparison to the standard chemotherapy arm at 26% (95% PI, 9–43%). Interestingly, the impact of low-dose veliparib was further dissected in the phase III BrighTNess trial [66]. TNBC patients were randomized in a three-arm study, to either receive veliparib plus carboplatin plus paclitaxel, carboplatin plus paclitaxel, or only paclitaxel, alongside the standard chemotherapy of doxorubicin and cyclophosphamide. There was no additional benefit of low-dose veliparib to the carboplatin plus paclitaxel backbone in terms of pCR (53% versus 58%; *p* = 0.357) or event-free survival (HR, 1.12; 95% CI, 0.72–1.72; *p* = 0.62) [17]. While it is easy to misinterpret these results as the lack of benefit of PARPi in combination with chemotherapy in early breast cancer [67], it is plausible that improved efficacy could be observed with an intermittent dosing schedule and a higher dose of veliparib at 120 mg twice daily, as shown in the BROCADE trials [62].

The efficacy of olaparib plus paclitaxel (OP) in comparison to carboplatin plus paclitaxel (CP) was evaluated in the GeparOLA trial, a randomized phase II study in early breast cancer patients in the neoadjuvant setting [68,69]. Olaparib was administered at 100 mg twice daily continuously, weekly paclitaxel at 80 mg/m2, and weekly carboplatin at AUC 2 for 12 weeks, all of which were followed by anthracycline-based chemotherapy. Eighty-six percent of patients were gBRCA^MUT^ and 73% of the cohort included TNBC patients. While the pCR rates were similar in the two groups, 55% for OP, in comparison to 49% in CP, grade 3/4 hematologic toxicities were less frequent in the OP arm (46.4%) versus the CT arm (78.4%) *p* = 0.002. The PARTNER trial tested olaparib at 150 mg twice daily for 12 days for four cycles in combination with carboplatin every 21 days and weekly paclitaxel for 4 cycles, followed by anthracycline-based chemotherapy in gBRCA^MUT^ or basal TNBC patients [69,70]. A preliminary pooled safety analysis demonstrated similar toxicities to conventional chemotherapy with the most common adverse events being neutropenia (19%), anemia (15%) and thrombocytopenia (5%). This is suggestive that a more potent PARPi can be used with either lower doses or an intermittent dosing schedule in combination with conventional chemotherapy with similar efficacy and possibly less toxicity than standard chemotherapy regimens.

### 3.2. PARPi in Combination with Immunotherapy

PARPi and immunotherapy have garnered much interest in TNBC and demonstrate great potential for synergy and improved overall survival in patients. The binding of PD-1 (on T-cells) by PD-L1 (on tumor cells) inhibits T-cell proliferation, cytokine release, and cytolytic activity, thereby restraining the immune response [71]. Compared to other breast cancer subtypes, TNBCs express elevated levels of PD-L1, which contributes to the evasion of tumor cells from immune-mediated destruction, rendering the treatment of such cancers challenging [72]. PD-1/PD-L1 blockade has been shown to potentiate anti-PARP therapy via attenuation of the cancer-associated immunosuppression through PD-L1 upregulation by PARPi [71]. Furthermore, PARPi efficacy may depend upon activation of the cGAS/STING pathway, which can lead to stimulation of antigen presentation by dendritic cells, resulting in CD8+T cell infiltration [73,74]. Additionally, macrophages have been shown to be the predominant tumor-infiltrating leukocyte in TNBC, creating an immunosuppressive microenvironment. Targeting such macrophages was shown to mitigate resistance to PARPi in BRCA^MUT^ TNBC [75]. Therefore, it is plausible that combining PARPi with immunotherapy may be an effective therapeutic approach in TNBC patients.

An open-labeled phase II single-arm study evaluated the combination of niraparib and pembrolizumab in metastatic TNBC patients [72]. Fifty-five patients had received ≤3 prior chemotherapy regimens. The ORR for all patients was 21%, (90% CI, 12–33%), but various factors were shown to enhance the ORR: BRCA^MUT^ (47% versus 11% BRCA^WT^ tumors); PD-L1-positive (32% versus 8% PD-L1-negative); and fewer lines of previous chemotherapy (27% for 0–1, versus 7% for 2–3). The MEDIOLA trial evaluated the combination of a PDL-1 inhibitor, durvalumab after a 4-week run-in of olaparib at 300 mg twice daily in 30 gBRCA^MUT^ metastatic breast cancer patients [76]. The ORR was 63% for all patients, and once again, the ORR increased to 78% amongst patients with no prior chemotherapy [77]. Despite the use of full doses of olaparib and durvalumab, there was an absence of dose-limiting toxicity. While several clinical trials are ongoing to investigate the utility of combining PARPi and immunotherapy agents [78], it is plausible that biomarker-based selection of patients may elicit the maximal potential of this combination.

### 3.3. PARPi in Combination with Targeted Therapies

The efficacy of PARPi has also been investigated in combination with various targeted therapies. For example, WEE1 kinase inhibitors (WEE1i) activate cyclin-dependent kinase (CDK)1 and CDK2 to regulate G1/S transition of the cell cycle and induce replication stress and DNA damage [79,80]. WEE1i were shown to overcome the G2 arrest induced by PARPi and induce mitotic catastrophe and cell death by apoptosis [80]. Indeed, the combination of WEE1i and PARPi was synergistic in 20/24 ovarian cancer cell lines. Interestingly, while concurrent WEE1i and PARPi administration resulted in poor in-vivo tolerance, sequential dosing of PARPi and WEE1i, improved toxicity while maintaining their efficacy [80]. Similarly, synthetic lethality with PARPi and ATM deficiency has also been identified. ATM deficiency was shown to induce DNA damage and increased PARylation. Therefore, the combination of an ATM inhibitor and PARPi led to extensive DNA damage, activating the G2 damage checkpoint kinase cascade, allowing entry into mitosis, and resulting in mitotic and post-mitotic cell death [81]. The inhibition of CHK1 kinase, which is a key component of checkpoint mediate cell cycle arrest, also demonstrated synergy with olaparib in basal-like breast cancer cells [82]. A phase I clinical trial further investigated the combination of CHK1 and olaparib and demonstrated preliminary antitumor activity in the context of BRCA^MUT^ patients with high-grade serous ovarian cancer with a previous progression on PARPi alone [83].

## 4. PARPi in Elderly Patients

A population of interest who may benefit from the use of PARPi are elderly patients. Aging is a risk factor for cancer with 44% of new breast and ovarian cancer cases, and 60–66% of breast and ovarian cancer-related deaths occurring in adults aged ≥65 [84]. In a large retrospective database study of TNBC patients aged 70 or older, 47% of patients received chemotherapy, 17% of patients were recommended chemotherapy but were not administered, and 36% of patients were not recommended chemotherapy [85]. Indeed, patients who received chemotherapy demonstrated a benefit in OS in both node-negative and node-positive tumors. Yet, age-specific data from clinical trials using PARPi is limited as older adults are frequently under-represented. Patients who are enrolled are highly selected with good performance status (ECOG 0-1) and adequate organ function, and thus are not representative of the patient characteristics from a real-world setting [84]. A pooled analysis of older adults that included eight phase 1–2 studies of olaparib in advanced recurrent ovarian cancer (N = 78) demonstrated that tolerability and toxicity are similar between patients aged <65 and ≥65 [86]. Indeed, further clinical trials are required to better evaluate PARPi in the elderly population. However, since PARPi are orally available, offer improved efficacy and quality of life with less toxicity in comparison to chemotherapy, have utility in BRCA^MUT^ and potentially BRCA^WT^ patients, it is conceivable that PARPi will play an important therapeutic role amongst elderly TNBC patients.

## 5. Predictive Biomarkers of Response to PARPi

Clinical efficacy of two PARPi in gBRCA^MUT^ breast cancer patients demonstrated an ORR of 60% in the metastatic setting, an improvement in invasive disease-free and distant disease-free survival in the adjuvant setting, and a pCR of ~50% in the neoadjuvant setting [26,27,52,53,54]. Although tested in smaller clinical cohorts, the efficacy of PARPi has also been identified in ~60% of unselected primary TNBC patients [55,56]. Preclinically, we and others have identified the efficacy of PARPi in TNBC cell line panels and patient-derived xenograft (PDX) models, regardless of *BRCA*-mutation status [87,88,89]. Indeed, the BRCAness of TNBCs needs to be better defined. Therefore, strong predictive biomarkers of therapeutic response to PARPi can be of great clinical utility to better select which TNBC patients can benefit from PARPi. Since there are few predictive biomarkers for PARPi that have been evaluated in clinical trials with TNBC patients, we will provide an overview of these trials and some of the predictive biomarkers evaluated in breast and other cancer types. We will be discussing biomarkers that are derived from homologous recombination defects (HRD) alone, such as *BRCA1/2* and other gene mutations, loss of heterozygosity, and genomic instability scores, in addition to gene expression signatures, that can incorporate several pathways that are not just dependent on HRD.

### 5.1. BRCA1/2 Mutations

The role of germline mutations as a strong predictive biomarker of PARPi response was demonstrated in two of the earlier studies with a greater ORR amongst BRCA^MUT^ patients versus BRCA^WT^ patients, 41% versus 24% respectively [47], and a longer PFS amongst BRCA^MUT^ (11.2 months for olaparib versus 4.3 months for placebo) in comparison to BRCA^WT^ patients (7.4 months for olaparib versus 5.5 months for placebo) [90]. However, both studies comprised heavily pretreated ovarian cancer patients, who either received a median of three prior chemotherapy regimens or at least two or more previous courses of platinum therapy with an objective response. This led to two recent phase III randomized controlled trials in gBRCA^MUT^ metastatic HER2-negative breast cancer patients, which demonstrated an ORR of approximately 60% [26,27].

While a meta-analysis of 18 studies suggested similar response rates for PARPi with germline and somatic *BRCA1/2* mutations [91], less is known about the role of somatic mutations of *BRCA1/2* in predicting response to PARPi in breast cancer. This may in part be due to the low frequency of deleterious somatic mutations of *BRCA1/2* of ~3% in all breast cancers [9], in comparison to 19% in ovarian cancers [92]. Nonetheless, the efficacy of PARPi has been demonstrated amongst breast cancer patients with somatic mutations in *BRCA1/2*, including a cohort of 16 metastatic patients, where an ORR of 50% was observed (90% CI, 28% to 72%) [51,93].

PARPi responses were demonstrated in both gBRCA1^MUT^ and gBRCA2^MUT^ patients, but controversy remains regarding which subgroup may confer greater sensitivity. Although a preclinical study identified a 2.3-fold enhanced sensitivity to PARPi in *BRCA2*-deficient in comparison to *BRCA1*-deficient cells [28], clinical trials have demonstrated mixed results. Indeed, a meta-analysis identified comparable efficacy of PARPi in BRCA1^MUT^ versus BRCA2^MUT^ solid cancers [94]. Since no trial has performed a back-to-back comparison of efficacy in BRCA1^MUT^ and BRCA2^MUT^ groups, we will describe the results of subgroup analysis from four randomized trials in breast cancer. In metastatic breast cancer, while olaparib was associated with an improved PFS in gBRCA1^MUT^ patients (HR, 0.54, 95% CI, 0.37–0.79) in comparison to gBRCA2^MUT^ patients (HR, 0.68; 95% CI, 0.45–1.07) [26], talazoparib was associated with an improved PFS in gBRCA2^MUT^ (HR, 0.49; 95% CI, 0.32–0.70) versus gBRCA1^MUT^ patients (HR, 0.59; 95% CI, 0.39–0.90) [27]. In combination with chemotherapy, veliparib demonstrated similar median PFS in the metastatic setting, regardless of mutation in either *BRCA1/2* [63]. In the adjuvant setting, no difference in invasive disease-free survival was observed with olaparib in either gBRCA1/2^MUT^ subgroup [52]. Therefore, BRCA1^MUT^ and BRCA2^MUT^ are probably equivalent in terms of predictive performance of PARPi response in breast cancer.

It is plausible that the PARPi efficacy observed in clinical trials may be limited in part due to primary (pre-existent/tumor intrinsic) or the development of acquired therapeutic resistance (decrease in treatment efficacy after initial tumor response). One mechanism that may explain primary and acquired resistance to DNA-damaging therapy (platinum-based or PARPi) amongst patients with germline or somatic mutations in *BRCA1/2* is the development of reversion mutations in *BRCA1/2* [95,96,97]. Usually, mutations in *BRCA1/2* are small insertion/deletions that result in a frameshift with a premature stop codon, which lead to a truncated, nonfunctional protein. Reversion or secondary mutations often lead to the conversion of the initial frameshift mutation into an in-frame internal deletion that still produces a partly functional protein, resulting in the restoration of efficient homologous recombination repair (HRR) [98,99].

*BRCA1/2* reversion mutations have been identified in smaller breast cancer cohorts with progressive disease on PARPi/platinum-based therapy, with a prevalence of ~40–50% of patients [100,101]. *BRCA1/2* reversion mutations were also identified retrospectively from circulating tumor DNA (ctDNA) in patients from the BROCADE3 trial (described above) and were part of the crossover arm, where they had received at least one dose of veliparib post-chemotherapy [102]. *BRCA1/2* reversion mutations were identified in 16% of these patients (4/28), for which cell-free DNA was available. The mean duration of veliparib monotherapy was 0.8 months for patients with *BRCA* reversion mutations, in comparison to 4.4 months for patients without reversion mutations. Indeed, reversion mutations are commonly associated with established PARPi resistance, and will need to be further evaluated as a predictive biomarker to change treatment decisions.

### 5.2. HRR Gene Mutations

Due to the efficacy of PARPi observed in patients without germline mutations in *BRCA1/2*, other germline and somatic mutations involved in the HRR pathway have been evaluated. In particular, mutations in *ATM*, *CDK12*, *CHEK2*, *HDAC2*, *PALB2*, *RAD51C*, and *RAD51D* were associated with PARPi response in prostate and ovarian cancers [103,104,105]. In metastatic breast cancer patients, germline *PALB2* mutations were associated with an improved ORR of 82% with a median PFS of 13.3 months [51]. However, the mutational frequency of 600 HRR genes in 1500 breast cancers was found to be ~15% [106]. Therefore, due to the relative rarity of individual non-*BRCA1/2* HRR gene mutations, such mutations would be difficult to use in the clinic as predictive biomarkers.

### 5.3. Copy Number Based “Genomic Scar” Assays

The deficiency of homologous recombination has also been shown to leave behind specific patterns of genomic alterations, called “genomic scars” [107,108]. In 2012, three independent groups reported a specific type of genomic scar using SNP array data [109]. The three types of genomic scars include: number of telomeric allelic imbalances (NtAI), which enumerates the subtelomeric regions with allelic imbalance extending from the centromere to telomere; large scale transitions (LST), which measures the number of chromosomal breaks between adjacent regions of at least 10 Mb; and homologous recombination deficiency-loss of heterozygosity (HRD-LOH), which counts the number of regions with LOH exceeding 15 Mb, but shorter than the whole chromosome.

These findings led to the development of two commercially available tests [110]: (1) genomic instability score (GIS) or HRD score (myChoice HRD Test, Myriad Genetics), which is derived from the unweighted sum of NtAI, LST and LOH; and (2) the fraction of subchromosomal segments (excluding chromosome 17—since LOH was observed in almost all samples of this chromosome in the discovery cohort) [111], called HRD-LOH score (FoundationFocus CDxBRCA, Foundation Medicine). HR deficiency, defined by an HRD score cutoff of 42, was evaluated retrospectively in three trials with TNBC patients treated with platinum-containing therapy in the neoadjuvant setting [112]. HR deficiency was predictive of RCB 0/1 both in univariate and multivariate models. However, the HRD score demonstrated different results in the context of randomized controlled trials. In the neoadjuvant GeparSixto trial, HR deficiency (cutoff of 42) was associated with pCR, (OR 2.60; 95% CI, 1.26–5.37; *p* = 0.008), but was not predictive of carboplatin benefit [113]. In the BrighTNess trial, although higher rates of pCR were observed amongst HR-deficient tumors, no differences were observed between any of the treatment arms including PARPi-based or platinum-based therapy, using cutoffs of 42 or 33 [114]. In a prospective neoadjuvant phase II study, no predictive association was identified between HRD score and pCR with either cisplatin or paclitaxel [115]. Furthermore, in metastatic randomized controlled trials, the HRD score was not predictive of response to either carboplatin in unselected TNBC patients [116], or olaparib amongst gBRCA^MUT^ breast cancer patients [117]. Overall, this is suggestive that the HRD score may be a prognostic biomarker, but further studies with a potent PARPi will be required to determine its predictive potential for PARPi response in TNBC.

Retrospective analysis of the HRD-LOH scores was also performed. In a single-arm study, HRD-LOH scores correlated with carboplatin-based therapeutic response amongst all TNBC patients and after exclusion of BRCA^MUT^ patients [118]. When LOH status was combined with LST, the mean values were able to discriminate between responders and non-responders to platinum therapy amongst BRCA^WT^ metastatic TNBC patients in another single-arm study [119]. Interestingly, in the OlympiAD trial with exclusively gBRCA^MUT^ patients, gene-specific LOH was identified in 94% of patients, suggesting a high rate of biallelic inactivation, but was not associated with response to olaparib [117]. The prognostic role of LOH was well demonstrated in BRCA^WT^ patients with ovarian cancer in the ARIEL 2 and ARIEL 3 trials [110]. Here, improvements in PFS were shown when comparing LOH-high versus LOH-low (HR 0.62; 95% CI, 0.42–0.90; *p* = 0.011), and when comparing rucaparib versus placebo amongst LOH-high patients (HR 0.29; 95% CI, 0.29–0.66; *p* < 0.0001) [120,121]. However, different cutpoints were used to define LOH-high in these studies, which also occurred with the HRD-score analysis [114], making clinical implementation in a prospective manner challenging. Furthermore, it is important to note that the commercial HRD tests were developed in platinum-sensitive cohorts of ovarian cancer patients [122], which is in contrast to the cohorts tested in breast cancer, comprising either TNBCs or mainly gBRCA^MUT^ populations, irrespective of prior platinum response.

### 5.4. Mutational Signatures

HRDetect was originally described to identify BRCAness in breast cancer. Using whole-genome sequencing, HRDetect was derived from a weighted algorithm of six genomic features, including the proportion of deletions at microhomology, the substitution signatures 3 and 8, the rearrangement signatures 3 and 5, and the copy-number derived HRD index scores [123]. HRDetect predicted HR deficiency with a sensitivity of 86–99% and was prevalent in up to 22% of all breast cancers using a cutoff of 0.7. Importantly, in TNBCs, 58.6% of tumors were identified as HRDetect-high (i.e., predictive of *BRCA1/2* deficiency). Amongst patients treated with chemotherapy, HRDetect was associated with invasive disease-free survival (HR, 0.42; 95% CI, 0.2–0.87) [124]. Furthermore, in a smaller cohort of 33 advanced-stage breast cancer patients treated with platinum, HRDetect was associated with OS (*p* = 0.04) [125]. In a cohort of 43 treatment-naïve TNBC patients, HRDetect identified 69% of patients as HR deficient [56]. Within this cohort, for which 15 patients were treated with rucaparib and had ctDNA levels available, an association was identified between HRDetect-positive and reduced ctDNA levels (*p* = 0.027). Altogether, HRDetect was identified in 59–69% of TNBC patients and has demonstrated promising results thus far. While further evaluation in larger cohorts will be required, the cost and feasibility of whole-genome sequencing, with utilization of fresh-frozen tissue [110], may be a hurdle for future clinical implementation.

### 5.5. Functional Biomarkers of HR Deficiency

A complementary approach to predict response to anti-PARP therapy is a functional assessment of the HR pathway [122,126]. Several studies have evaluated the role of RAD51, the main HRR recombinase, which is regulated by several proteins, including BRCA2, RAD52, PALB2, BRCA1, ATR, and ATM. Double-strand breaks in the G/S2 phase or stalled DNA replication forks can result in the formation of RAD51 foci. Foci are multiprotein complexes that organize around double-strand breaks and are visualized within the nuclei by immunofluorescence (IF) microscopy. While earlier studies evaluated RAD51 foci in response to chemotherapy or radiation therapy, more recent studies have assessed RAD51 foci in untreated tumors.

In 2010, Graeser et al. evaluated RAD51 focus formation in a cohort of 68 breast cancer patients from core biopsy tumor samples at 24 h post- neoadjuvant anthracycline-based chemotherapy [127]. A low RAD51 score (indicating HR deficiency) was present in 26% of all breast cancer patients and 67% of TNBC patients. Low RAD51 scores were associated with pCR (33% versus 3% in non-pCR tumors, *p* = 0.011). However, when considering the kinetics of the DNA damage response, it is plausible that a proportion of HR deficient tumors are not being captured at the 24-h time point [128]. Alternatively, using an ex-vivo assay, surgical breast samples were collected to study the impact of ionizing radiation upon RAD51 foci formation [128]. Low RAD51 scores were identified in 11% of all samples, of which one tumor was associated with PARPi response.

Contrary to previous studies which demonstrated low levels of endogenous DNA damage, recent studies have identified higher levels of DNA damage and detected RAD51 foci in untreated samples [129,130,131]. In a cohort of 13 BRCA^MUT^ breast cancer PDXs treated with olaparib, the baseline proportion of RAD51-high cells was higher amongst PARPi-sensitive tumors in comparison to PARPi-resistant tumors (24% versus 3%, *p* = 0.0025) [129]. In the RIO trial, HR deficiency from RAD51-IF was observed in 47% of samples (N = 17) [130]. Tumors with low RAD51 scores were associated with greater reductions in ctDNA levels when treated with rucaparib. In the GeparSixto trial, RAD51 scores were also shown to be concordant with HRD scores in a cohort of TNBC patients in the neoadjuvant setting [131]. Low RAD51 scores were identified in 61% of untreated TNBC patients and were associated with pCR in patients who received carboplatin-based therapy in comparison to paclitaxel plus a doxorubicin formulation (66% versus 33%, OR, 3.96, 2.56–20.05, *p* = 0.004; interaction test *p* = 0.02). Although further studies in larger cohorts are also required, some of the limitations of the RAD51 assay are that mediators of HRD downstream to RAD51 are not incorporated [110], and the presence of RAD51 foci does not reflect all mechanisms of double-strand break repair, but only HRR [126].

### 5.6. Gene Expression Signatures

Transcriptional signatures are advantageous as they can assess the modulation of a constellation of genes with a commonly performed approach of gene expression analysis. Furthermore, gene expression signatures can integrate different pathways, and not just HRD, which can influence response to PARPi [132].

Several gene signatures have been derived to predict response to PARPi [87,88,133,134]. McGrail et al. used a panel of solid cancer gene expression data with IC50 values from the GDSC (Genomics of Drug Sensitivity in Cancer) [133,135]. In two distinct datasets, 3-day cell viability assays were used with a maximum concentration of 5 μM, and at least 96% of cell lines demonstrated IC50 values >5 μM. The PARPi gene signature predicted response with high accuracy in a large panel of breast and ovarian cancer cell lines, in addition to three PDX tumors.

Daemen et al. identified a 7-gene signature (PARPi7), indicating a deficiency in DNA repair, which was derived from a panel of 22 ER-positive and ER-negative breast cancer cell lines, that was associated with response to olaparib [88]. The PARPi7 gene signature was detected in 8–21% of all breast cancers. When PARPi7 was combined with the 70-gene signature, MammaPrint (MP) High 1/High 2 risk status (MP1/2), the combined signature was present in 42% of TNBC patients in the I-SPY 2 TRIAL [136]. PARPi7 plus MP1/2 was associated with a pCR rate of 75% in patients treated with the combination of veliparib plus carboplatin. In addition, a 77-gene BRCA1ness expression signature was developed from whole-genome gene expression data from TNBC patients [133]. Since *BRCA1* mutation or promoter methylation can induce a specific copy number pattern, copy number profiles were used to classify the tumors into BRCA1-like versus non-BRCA1-like. In the discovery cohort, 48% of the cohort was identified as BRCA1-like, and significant pathways associated with the signature included cellular assembly and control, DNA replication, recombination, and repair, serine and glycine biosynthesis, and cell cycle control. In the I-SPY 2 TRIAL, the BRCA1ness signature was associated with pCR in the patients treated with the combination of veliparib plus carboplatin (*p* = 0.03), but not the control arm (*p* = 0.45). Since the combination of low-dose veliparib plus carboplatin did not demonstrate any additional benefit of PARPi [66], these gene signatures effectively demonstrated their predictive potential of carboplatin response.

We previously described a novel approach to identify a 63-gene signature associated with PARPi response [40,87]. Using a panel of 8 TNBC cell lines, we identified the efficacy of three PARPi, veliparib, olaparib, and talazoparib, by performing high-content imaging, measuring cell count, and calculating IC50 values. We also quantified the 53BP1 response post-treatment, a marker of double-strand breaks and DNA damage using single-cell analysis. We calculated EC50 values, the concentration required for 50% of maximal 53BP1 response, and found a strong correlation between EC50 and IC50 values.

Therefore, we used a functional readout of PARPi response, the 53BP1 response profile to 9 concentrations of each PARPi for each cell line to categorize our cell lines as sensitive or resistant. We used whole-transcriptome data from untreated cell lines to create a rank list, and a curated gene list associated with *BRCA1/2* mutation status, HRD, PARPi sensitivity, and DNA damage response to perform a gene set enrichment analysis, which yielded 176 genes. Using Reactome Enrichment Pathway Analysis, we then identified statistically significant pathways and 63 associated genes. In addition to HR, enriched pathways in cell-cycle checkpoints, base and nucleotide excision repair, and DNA damage bypass were identified.

Subsequently, we interrogated our combined PARPi 63-gene signature in a previously published cohort of 7 PDX tumors that were treated with olaparib. We compared the performance of our gene signature with 7 other gene sets that were either associated with PARPi response, HRD, BRCAness, or *BRCA1/2* mutation status. Although this was a small dataset, our 63-gene signature outperformed all other gene sets, with the highest overall accuracy of 86%. Moreover, we determined that our 63-gene signature predicted PARPi sensitivity in 45% of untreated TNBC patients.

While our 63-gene signature requires further validation in clinical cohorts treated with PARPi, our gene signature is unique in that it combined the efficacy of three PARPi, used the DNA damage response to categorize response, and identified several implicated pathways specific to PARPi response. With the potential to identify ~50% of all TNBC patients, including BRCA^MUT^ and BRCA^WT^, our 63-gene signature offers great potential to select a patient subpopulation that can benefit from PARPi.

## 6. Conclusions

Over the past decade, several studies have brought into perspective the current and potential clinical utility of PARPi in TNBC. Clearly, initial enthusiasm about PARPi in breast cancer may have been dampened due to limited efficacy in heavily pre-treated cohorts. However, repeatedly, in studies using PARPi in monotherapy or combination, improved efficacy was observed in cohorts who received no or very little prior chemotherapy regimens. This is suggestive that PARPi may demonstrate their true potential in either the neoadjuvant or adjuvant settings, both of which have been the least studied contexts to date.

Studies have also started to demonstrate the efficacy of PARPi beyond gBRCA^MUT^ status, amongst TNBC who are chemotherapy-naïve. While several predictive biomarkers have been evaluated, most of these are surrogate markers of HRD, which may not reflect the interactions of different pathways involved in modulating PARPi response. Gene expression signatures can integrate different pathways and can be easily implemented for clinical use. It is also important to note that there is precedence that biomarkers used in one clinical context may not necessarily be the same in another clinical context for the same cancer subtype. For example, improved outcomes of atezolizumab and pembrolizumb were demonstrated in PD-L1+ tumors in the metastatic setting, but pembrolizumab was associated with improved pCR and event-free survival in all TNBCs in the neoadjuvant setting, regardless of PDL-1 status [21,23,137]. Therefore, it is possible that while *BRCA1/2* mutation status may play an important role in predicting PARPi response in metastatic patients, it may not necessarily have the same significance in early breast cancer patients.

Trials evaluating PARPi in combination have essayed PARPi of different potencies and varying doses, dosing schedules, and sequencing strategies. Veliparib, a low potency PARPi, was only found to provide an added benefit to standard chemotherapy at higher doses and with an intermittent dosing schedule. Efficacy with olaparib was also demonstrated with either an intermittent dosing schedule and pre-treatment with carboplatin or continuous lower doses of olaparib. Toxicity was a significant concern with continuous and concomitant dosing of talazoparib in combination with carboplatin. However, it will be interesting to observe the efficacy and toxicity of the newly developed selective PARP-1 inhibitor that has demonstrated promising preclinical results and is currently in a Phase I trial [138].

**Figure 2:**
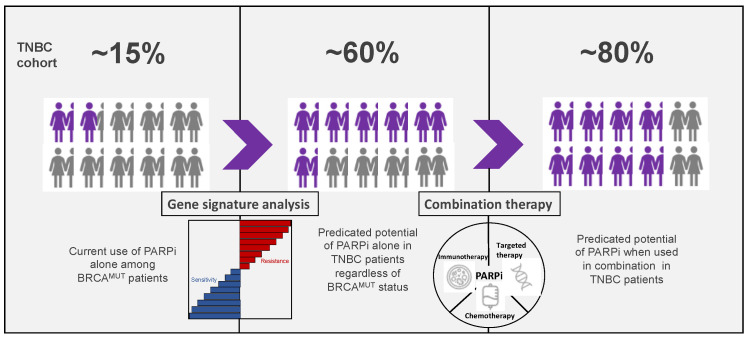
Projected utility of PARPi in TNBC. Currently, PARPi are used only among germline, *BRCA1/2*-mutant metastatic TNBC patients, which constitute about 15% of TNBC patients. With the use of strong predictive biomarkers in early TNBC, it is plausible that PARPi sensitivity will be observed in about 60% of TNBC patients (middle panel). In combination with either chemotherapy, immunotherapy, or targeted therapeutics, PARPi sensitivity can potentially increase to 80% of TNBC patients (right panel).

While larger and randomized controlled trials are required to further elucidate the role of PARPi in TNBC, we envision an expanding role of PARPi in TNBC (Figure 2). Since two studies with untreated primary breast cancer demonstrated a PARPi response in 56–68% of TNBC patients, and four predictive biomarkers (HRDetect, baseline RAD51 foci, BRCA1ness, and 63-gene signature) predicted BRCAness in 45–69% of TNBCs, it is highly probable that PARPi can be effective as monotherapy in about ~60% of early TNBCs. Furthermore, since the ORR of olaparib or talazoparib was ~60% as monotherapy in gBRCA^MUT^ metastatic breast cancer patients, and an ORR of 80–88% were observed when veliparib or olaparib was administered in combination with chemotherapy, we envision that combination approaches could add a benefit of similar magnitude in early TNBC patients. In this manner, PARPi in combination may be an effective and less toxic approach to de-escalate chemotherapy in patients with early TNBC. Therefore, while there has been much progress in the use of PARPi in breast cancer thus far, there is great potential for further use in newer clinical contexts for TNBC patients.

## Figures and Tables

**Table 2:** Clinical trials with PARP inhibitors in combination with chemotherapy in the metastatic setting.

First Author Study Name	Year of Study	No. of Participants (BC Patients)	Type of Study	Median/Mean No. of Prior Chemotherapy Regimens (Range)	Therapeutic Agents			Patient Population	Outcome (Objective Response Rate (ORR), Progression Free Survival (PFS))
					PARPi	Combination Agent	Comparative Agent/ Standard Chemotherapy		
Lee, J.M. et al. [57]	2014	45 (8 BC)	Phase I/Ib	5 (2–11)	Olaparib capsule, 100 mg twice daily continuous or Olaparib capsule, 200–400 mg twice daily days 1–7	Carboplatin AUC 3–5 every 21 days	None	gBRCA^MUT^	ORR in all 52.4%, ORR in breast cancer 87.5%
Lee, J.M. et al. [58]	2017	77 (14 BC)	Phase I/Ib	4 (1–10)	Dose escalation: Olaparib tablet: 100–200 mg twice daily, days 1–7 300 mg twice daily maintenance after carboplatin Expansion cohort: Olaparib: Cohort A: Days 1–7 cycle 1, and days 2–8 for cycle 2; Cohort B: Days 2–8 cycle 1, and 1–7 cycle 2. Both cohorts: Days 1–7 cycle 3 up to 8; olaparib maintenance	Dose escalation: Carboplatin AUC4–5 every 21 days, up to 8 cycles Expansion cohort: Carboplatin: Cohort A: Day 8 cycle 1, day 1 cycle 2; Cohort B: Day 1 cycle 1, day 8 cycle 2 Both cohorts: Day 1 cycle 3, up to 8	None	Recurrent or refractory gynecologic cancers or metastatic or inoperable breast cancer	ORR in all 46%, gBRCA^MUT^ 68%
Dhawan, M.S. et al. [59]	2017	24 (11 BC)	Phase I	24% ≤ 2 lines * 12%—3 lines * 63% ≥ 4 lines*	Talazoparib 0.75 and 1 mg daily	Carboplatin AUC 1 and 1.5 every 2–3 weeks	None	Advanced solid tumors	ORR in all 14%
Somlo, G. et al. [60]	2017	77 (77 BC)	Phase I/II	Phase I: 1 (0–5) Phase II: 1 (0–5)	Phase 1: Veliparib, 50–200 mg twice daily Phase 2: Veliparib, 400 mg twice daily and upon progression 150 mg twice daily in combination	Phase 1: Carboplatin AUC 5/6 every 21 days Phase 2: Carboplatin AUC 5 every 21 days in combination	None	gBRCA^MUT^ breast cancer	Response rate in phase I, 56%; phase II—BRCA1^MUT^, 14%; BRCA2^MUT^, 36%, PFS in phase I, 8.7 months; phase II—on veliparib, 5.2 months; after combination therapy, 1.8 months
Appleman, L.J. et al. [61]	2019	73 (16 BC)	Phase I	≤3 lines	Veliparib 10–120 mg twice daily Days 1–7, starting cycle 2	Carboplatin: AUC 6 Paclitaxel: 150–200 mg/m^2^ Day 1 of 21-day cycle 1, Day 3 of cycle 2 onwards	None	Advanced solid tumors	ORR in all 40%, ORR in breast cancer 69%
Han, H.S. et al. [62] BROCADE	2018	294 (294 BC)	Phase II randomized controlled trial	≤2 lines	Veliparib (V) 120 mg twice daily Days 1–7, 21-day cycles	Carboplatin (C): AUC 6 Paclitaxel (P): 75 mg/m^2^ Day 3	PCP (placebo, carboplatin, paclitaxel) vs. V plus temozolomide (T)	gBRCA^MUT^ breast cancer	ORR in VCP, 77.8%, PCP, 61.3%; VT, 28.6% PFS in VCP, 14.1 months; CP 12.3 months, V plus T, 7.4 months
Diéras, V. et al. [63] Arun, B.K. et al. [64] BROCADE3	2020	513 (513 BC)	Phase III Double-blinded, randomized controlled trial	≤2 lines	Veliparib, 120 mg twice daily Days −2 to 5 If combination discontinued prior to progression, could continue with veliparib up to 400 mg twice daily	Carboplatin (C) AUC 6 Day 1 of 21-day cycle Paclitaxel (P) 80 mg/m^2^ Day 1, 8, 15 of 21-day cycle	PCP (placebo, carboplatin, paclitaxel)	gBRCA^MUT^ HER2-negative breast cancer	All ORR in VCP 75.8%, PCP 74.1% PFS in VCP 14.5 months, PCP 12.6 months No previous chemotherapy ORR in VCP 79.7%, PCP 76.3% PFS in VCP 16.6 months, PCP 13.1 months

Abbreviations: BC, breast cancer. * Previous treatment regimens.

## Data Availability

Data sharing not applicable.
